# Supplementary material for: Clinical and immunological outcomes of HIV-exposed uninfected and HIV-unexposed uninfected children in the first 24 months of life in Western Kenya
Source: BMC Infect Dis. 2024 Feb 1;24:156. doi: 10.1186/s12879-024-09051-3 (PMC10835872; doi:10.1186/s12879-024-09051-3)
Supplement: Supplementary file 1 — Additional file 1: Supplementary Table 1. Sources of recombinant P. falciparum protein antigens, protein concentrations used in conjugation to magnetic microspheres, expression systems, and salient references. Supplementary Table 2. Birth characteristics of HEU and HUU infants. Mean gestational age, birth weight, length, and head circumference were compared between the two groups using the Student’s t-test; sd, standard deviation. Supplementary Table 3. Linear growth curve models of Z scores for weight, length, head circumference, and BMI for age in HEU vs. HUU children, obtained using Bayesian hierarchical regression. HDI, highest probability density interval; sd, standard deviation. Region of practical equivalence (ROPE) for contrast terms for intercepts and slopes set at -0.05, 0.05. Supplementary Table 4. Fixed effects regression models for plasma cytokine levels. Twelve cytokines were measured at 8 visits (birth, 6, 10, 14, 18, 26, 39, and 52 weeks of age). A total of 663 visits were analyzed. Differences between HEU and HUU children in plasma cytokine trajectories were tested by evaluating the group*time interaction effect. If there was no difference, we tested whether HEU values differed from HUU values (group main effect) and whether values changed over time within a group (time main effect) (of interest was the 26 weeks vs. birth comparison, which is included in the table). Supplementary Table 5. Fixed effects regression models for vaccine-specific antibody levels. Antibodies against four vaccines were measured at 12 visits (birth, 6, 10, 14, 18 weeks, 6, 9, 12, 15, 18, 21, and 24 months of age). A total of 782 visits were analyzed. Differences between HEU and HUU children in vaccine antibody trajectories were tested by evaluating the group*time interaction effect. We then tested whether HEU values differed from HUU values (group main effect) and whether values changed over time within a group (time main effect) (of interest were the 6 months vs. birth and [file 12879_2024_9051_MOESM1_ESM.docx]

**Supplementary Material**

**Supplementary Table 1.** Sources of recombinant *P. falciparum* protein antigens, protein concentrations used in conjugation to magnetic microspheres, expression systems, and salient references.

| **Pf Antigen** | **Abbreviation** | **Concentration (μg per 6.125 x 10^5^ beads)** | **Expression System** | **Provider** | **Ref.** |
| --- | --- | --- | --- | --- | --- |
| Circumsporozoite protein | CSP | 0.25 | *E. coli* | Sheetij Dutta | [1] |
| Apical membrane antigen 1 | AMA-1 3D7 | 0.30 | *Pichia pastoris* | David Narum | [2] |
| Erythrocyte binding antigen 140 | EBA-140 | 0.25 | *E. coli* | Alan Cowman | [3] |
| Erythrocyte binding antigen 175 | EBA-175 3D7 | 0.08 | *E. coli* | Alan Cowman | [4] |
| Erythrocyte binding antigen 181 | EBA-181 | 0.10 | *E. coli* | Alan Cowman | [5] |
| Merozoite surface protein 1 | MSP1 (42) FVO | 0.20 | *E. coli* | Evelina Angov | [6] |
| Merozoite surface protein 2 | MSP2 Fc27 | 0.10 | *E. coli* | James Beeson | [7] |
| Merozoite surface protein 3 | MSP3 | 0.50 | *E. coli* | David Narum | [8] |
| Merozoite surface protein 6 | MSP6 | 0.30 | *E. coli* | James Beeson | [9] |
| Merozoite surface protein 7 | MSP7 | 0.30 | *E. coli* | James Beeson | [10] |
| Merozoite surface protein 9 | MSP9 | 0.50 | *E. coli* | James Beeson | [10] |
| Merozoite surface protein Duffy binding-like 1 | MSP DBL1 | 0.50 | *E. coli* | Alan Cowman | [11] |
| Merozoite surface protein Duffy binding-like 2 | MSP DBL2 | 0.50 | *E. coli* | Alan Cowman | [11] |
| Reticulocyte binding protein homologue 5 | RH5 | 1.00 | *E. coli* | Alan Cowman | [12] |

**References for Supplementary Table 1**

1. Porter MD, Nicki J, Pool CD, et al. Transgenic parasites stably expressing full-length Plasmodium falciparum circumsporozoite protein as a model for vaccine down-selection in mice using sterile protection as an endpoint. Clin Vaccine Immunol **2013**; 20:803-10.

2. Dutta S, Lalitha PV, Ware LA, et al. Purification, characterization, and immunogenicity of the refolded ectodomain of the Plasmodium falciparum apical membrane antigen 1 expressed in Escherichia coli. Infect Immun **2002**; 70:3101-10.

3. Thompson JK, Triglia T, Reed MB, Cowman AF. A novel ligand from Plasmodium falciparum that binds to a sialic acid-containing receptor on the surface of human erythrocytes. Mol Microbiol **2001**; 41:47-58.

4. Reed MB, Caruana SR, Batchelor AH, Thompson JK, Crabb BS, Cowman AF. Targeted disruption of an erythrocyte binding antigen in Plasmodium falciparum is associated with a switch toward a sialic acid-independent pathway of invasion. Proc Natl Acad Sci U S A **2000**; 97:7509-14.

5. Gilberger TW, Thompson JK, Triglia T, Good RT, Duraisingh MT, Cowman AF. A novel erythrocyte binding antigen-175 paralogue from Plasmodium falciparum defines a new trypsin-resistant receptor on human erythrocytes. J Biol Chem **2003**; 278:14480-6.

6. Darko CA, Angov E, Collins WE, et al. The clinical-grade 42-kilodalton fragment of merozoite surface protein 1 of Plasmodium falciparum strain FVO expressed in Escherichia coli protects Aotus nancymai against challenge with homologous erythrocytic-stage parasites. Infect Immun **2005**; 73:287-97.

7. Reddy SB, Anders RF, Beeson JG, et al. High affinity antibodies to Plasmodium falciparum merozoite antigens are associated with protection from malaria. PloS one **2012**; 7:e32242.

8. Tsai CW, Duggan PF, Jin AJ, et al. Characterization of a protective Escherichia coli-expressed Plasmodium falciparum merozoite surface protein 3 indicates a non-linear, multi-domain structure. Mol Biochem Parasitol **2009**; 164:45-56.

9. Pearce JA, Triglia T, Hodder AN, Jackson DC, Cowman AF, Anders RF. Plasmodium falciparum merozoite surface protein 6 is a dimorphic antigen. Infect Immun **2004**; 72:2321-8.

10. Richards JS, Arumugam TU, Reiling L, et al. Identification and prioritization of merozoite antigens as targets of protective human immunity to Plasmodium falciparum malaria for vaccine and biomarker development. J Immunol **2013**; 191:795-809.

11. Lin CS, Uboldi AD, Marapana D, et al. The merozoite surface protein 1 complex is a platform for binding to human erythrocytes by Plasmodium falciparum. J Biol Chem **2014**; 289:25655-69.

12. Reddy KS, Pandey AK, Singh H, et al. Bacterially expressed full-length recombinant Plasmodium falciparum RH5 protein binds erythrocytes and elicits potent strain-transcending parasite-neutralizing antibodies. Infect Immun **2014**; 82:152-64.

**Supplementary Table 2:** Birth characteristics of HEU and HUU infants. Mean gestational age, birth weight, length, and head circumference were compared between the two groups using the Student’s t-test; sd, standard deviation.

|  | HEU | HUU | *p* value |
| --- | --- | --- | --- |
| No. | 82 | 169 |  |
| Female (%) | 40 (49.5) | 81 (48.5) |  |
| Gestational age (wk), mean (sd) | 37.2 (4.4) | 38.2 (3.8) | 0.39 |
| Weight (kg), mean (sd) | 3.2 (0.52) | 3.2 (0.51) | 0.68 |
| Length (cm), mean (sd) | 47.5 (2.69) | 47.7 (3.08) | 0.53 |
| Head circumference, mean (sd) | 34.6 (1.97) | 35.1 (1.86) | 0.07 |

**Supplementary Table 3:** Linear growth curve models of Z scores for weight, length, head circumference, and BMI for age in HEU vs. HUU children, obtained using Bayesian hierarchical regression. HDI, highest probability density interval; sd, standard deviation. Region of practical equivalence (ROPE) for contrast terms for intercepts and slopes set at -0.05, 0.05.

|  | | **Posterior Mean** | **95% HDI** | $\hat{\boldsymbol{R}}$ |
| --- | --- | --- | --- | --- |
| **Weight for Age Z Scores** | | | | |
|  | HUU:HEU intercept | 0.1549 | -0.1197, 0.4522 | 1.0001 |
|  | HUU:HEU slope | -0.0023 | -0.0191, 0.0147 | 1.0004 |
|  | sd intercept | 0.9851 | 0.8805, 1.0959 | 1.0023 |
|  | sd slope | 0.0413 | 0.0333, 0.0492 | 1.0015 |
| **Length for Age Z Scores** | | | | |
|  | HUU:HEU intercept | -0.1277 | -0.4755, 0.2131 | 1.0000 |
|  | HUU:HEU slope | -0.0172 | -0.0414, 0.0078 | 1.0000 |
|  | sd intercept | 1.0651 | 0.9222, 1.2132 | 1.0002 |
|  | sd slope | 0.0533 | 0.0396, 0.0670 | 1.0011 |
| **Head Circumference for Age Z Scores** | | | | |
|  | HUU:HEU intercept | 0.0407 | -0.2788, 0.3666 | 1.0000 |
|  | HUU:HEU slope | -0.0047 | -0.0247, 0.0150 | 1.0000 |
|  | sd intercept | 1.0494 | 0.9211, 1.1769 | 1.0001 |
|  | sd slope | 0.0444 | 0.0335, 0.0554 | 1.0004 |
| **BMI for Age Z Scores** | | | | |
|  | HUU:HEU intercept | 0.9107 | -0.0375, 0.6810 | 1.0551 |
|  | HUU:HEU slope | -0.0297 | -0.0153, 0.0369 | 1.0490 |
|  | sd intercept | 1.2230 | 0.6609, 1.0144 | 1.0530 |
|  | sd slope | 0.0624 | 0.0098, 0.0534 | 1.0377 |

**Supplementary Table 4:** Fixed effects regression models for plasma cytokine levels. Twelve cytokines were measured at 8 visits (birth, 6, 10, 14, 18, 26, 39, and 52 weeks of age). A total of 663 visits were analyzed. Differences between HEU and HUU children in plasma cytokine trajectories were tested by evaluating the group*time interaction effect. If there was no difference, we tested whether HEU values differed from HUU values (group main effect) and whether values changed over time within a group (time main effect) (of interest was the 26 weeks vs. birth comparison, which is included in the table).

| Interaction effect (Group*Time) | | Num DF | Den DF | F value | Pr > F |
| --- | --- | --- | --- | --- | --- |
| IFNg | | 7 | 532 | 0.93 | 0.48 |
| IL-1b | | 7 | 531 | 1.04 | 0.4 |
| IL-6 | | 7 | 524 | 1.51 | 0.16 |
| IL-10 | | 7 | 532 | 0.91 | 0.5 |
| IL-12p70 | | 7 | 532 | 0.6 | 0.75 |
| IL-17A | | 7 | 532 | 0.78 | 0.6 |
| IL-17E | | 7 | 532 | 0.71 | 0.66 |
| IL-17F | | 7 | 530 | 0.72 | 0.66 |
| IL-21 | | 7 | 532 | 0.78 | 0.6 |
| IL-22 | | 7 | 530 | 0.79 | 0.59 |
| IL-23 | | 7 | 532 | 0.65 | 0.71 |
| TNF | | 7 | 532 | 1.32 | 0.24 |
| Group main effect (HEU vs. HUU) | **Estimate** | **Std error** | **DF** | **t Value** | **Pr > \|t\|** |
| IFNg | -4.43 | 33.15 | 115 | -0.13 | 0.89 |
| IL-1b | 5.93 | 15.00 | 115 | 0.4 | 0.69 |
| IL-6 | 1.98 | 9.42 | 115 | 0.21 | 0.83 |
| IL-10 | -3.02 | 6.50 | 115 | -0.46 | 0.64 |
| IL-12p70 | 10.25 | 28.57 | 115 | 0.36 | 0.72 |
| IL-17A | 11.34 | 17.73 | 115 | 0.64 | 0.52 |
| IL-17E | -0.95 | 0.61 | 115 | -1.57 | 0.12 |
| IL-17F | 0.005 | 0.02 | 115 | 0.2 | 0.84 |
| IL-21 | -17.94 | 46.79 | 115 | -0.38 | 0.7 |
| IL-22 | -0.79 | 0.32 | 115 | -2.5 | **0.01** |
| IL-23 | -3.19 | 6.26 | 115 | -0.51 | 0.61 |
| TNF | 17.39 | 17.93 | 115 | 0.97 | 0.33 |
| Time main effect (26 wks vs. birth) | **Estimate** | **Std error** | **DF** | **t Value** | **Pr > \|t\|** |
| IFNg | 167.18 | 44.55 | 539 | 2.41 | **0.02** |
| IL-1b | 62.83 | 19.35 | 538 | 3.25 | **0.001** |
| IL-6 | 9.52 | 12.80 | 531 | 0.74 | 0.46 |
| IL-10 | 43.45 | 9.07 | 539 | 4.79 | **<0.0001** |
| IL-12p70 | 132.98 | 36.50 | 539 | 3.64 | **0.0003** |
| IL-17A | 84.34 | 22.78 | 539 | 3.7 | **0.0002** |
| IL-17E | 2.87 | 0.78 | 539 | 3.68 | **0.0003** |
| IL-17F | 0.09 | 0.03 | 537 | 2.68 | **0.008** |
| IL-21 | 229.69 | 59.04 | 539 | 3.89 | **0.0001** |
| IL-22 | 1.39 | 0.42 | 537 | 3.32 | **0.001** |
| IL-23 | 30.71 | 8.02 | 539 | 3.83 | **0.0001** |
| TNF | 87.54 | 23.60 | 539 | 3.71 | **0.0002** |

**Supplementary Table 5:** Fixed effects regression models for vaccine-specific antibody levels. Antibodies against four vaccines were measured at 12 visits (birth, 6, 10, 14, 18 weeks, 6, 9, 12, 15, 18, 21, and 24 months of age). A total of 782 visits were analyzed. Differences between HEU and HUU children in vaccine antibody trajectories were tested by evaluating the group*time interaction effect. We then tested whether HEU values differed from HUU values (group main effect) and whether values changed over time within a group (time main effect) (of interest were the 6 months vs. birth and 18 months vs. 6 months comparisons, which are included in the table).

| Interaction effect (Group*Time) | | Num DF | Den DF | F value | Pr > F |
| --- | --- | --- | --- | --- | --- |
| Diphtheria | | 11 | 643 | 0.54 | 0.88 |
| Tetanus | | 11 | 620 | 1.3 | 0.22 |
| Hepatitis B | | 11 | 645 | 0.64 | 0.8 |
| Measles | | 11 | 644 | 1.97 | **0.03** |
| Group main effect (HEU vs. HUU) | **Estimate** | **Std error** | **DF** | **t Value** | **Pr > \|t\|** |
| Diphtheria | 0.042 | 0.044 | 113 | 0.96 | 0.34 |
| Tetanus | -0.34 | 0.21 | 102 | -1.62 | 0.11 |
| Hepatitis B | 0.63 | 0.71 | 113 | 0.89 | 0.37 |
| Measles | 0.09 | 0.15 | 113 | 0.64 | 0.52 |
| Time main effect  (6 mo vs. birth) | **Estimate** | **Std error** | **DF** | **t Value** | **Pr > \|t\|** |
| Diphtheria | -0.43 | 0.05 | 654 | -8.61 | **<0.0001** |
| Tetanus | 1.35 | 0.22 | 631 | 6.03 | **<0.0001** |
| Hepatitis B | -9.96 | 1.03 | 656 | -9.63 | **<0.0001** |
| Measles | 1.02 | 0.199 | 655 | 5.09 | **<0.0001** |
| Time main effect (18 mo vs. 6 mo) | **Estimate** | **Std error** | **DF** | **t Value** | **Pr > \|t\|** |
| Diphtheria | -0.33 | 0.06 | 654 | -5.91 | **<0.0001** |
| Tetanus | -1.23 | 0.22 | 631 | -5.64 | **<0.0001** |
| Hepatitis B | -9.17 | 1.18 | 656 | -7.8 | **<0.0001** |
| Measles | 2.03 | 0.22 | 655 | 9.04 | **<0.0001** |

**Supplementary Table 6:** Fixed effects regression models for antimalarial antibody levels (interaction effects and main effects for group). Antibodies against 14 *P. falciparum* antigens were measured at 12 visits (birth, 6, 10, 14, 18 weeks, 6, 9, 12, 15, 18, 21, and 24 months of age). A total of 1,135 visits were analyzed. Differences between HEU and HUU children in antimalarial antibody trajectories were tested by evaluating group*time interaction effect. For 12 of 14 antigens, we then tested whether HEU values differed from HUU values (group main effect); mean antibody values (fold over North American controls) of the group averaged over all time points are listed.

|  | Interaction effect (Group*Time) | Group main effect | | |
| --- | --- | --- | --- | --- |
|  | ***p* value** | **HUU** | **HEU** | ***p* value** |
| CSP | 0.06 | 3.8 | 2.2 | **0.008** |
| AMA-1 3D7 | 0.001 | 47.3 | 31.9 | **0.002** |
| EBA-140 | 0.14 | 5.1 | 4.2 | 0.33 |
| EBA-175 | 0.66 | 7.9 | 7.2 | 0.68 |
| EBA-181 | 0.14 | 4.8 | 2.8 | 0.11 |
| MSP1 | 0.19 | 27.5 | 28.2 | 0.86 |
| MSP2 | **0.01** |  |  |  |
| MSP3 | 0.14 | 5.6 | 2.7 | **0.005** |
| MSP6 | **0.0002** |  |  |  |
| MSP7 | 0.85 | 5.6 | 2.2 | 0.06 |
| MSP9 | 0.08 | 5.5 | 4 | **0.009** |
| MSP DBL1 | 0.02 | 18.1 | 11.4 | **0.004** |
| MSP DBL2 | 0.22 | 28.3 | 21.2 | 0.15 |
| Rh5 | 0.6 | 2.1 | 2.6 | 0.37 |

**Supplementary Table 7:** Fixed effects regression models for antimalarial antibody levels (main effects for time). Antibodies against 14 *P. falciparum* antigens were measured at 12 visits (birth, 6, 10, 14, 18 weeks, 6, 9, 12, 15, 18, 21, and 24 months of age). A total of 1,135 visits were analyzed. For 12 of 14 antigens, we tested whether values changed over time independent of group. Of interest were 6 months vs. birth and 18 months vs. 6 months comparisons. Mean antibody values (fold over North American controls) at the three time points are listed.

| Antigen | Birth (mean) | 6 mo (mean) | 18 mo (mean) | Birth vs. 6 mo  *p* value | 6 mo vs. 18 mo *p* value |
| --- | --- | --- | --- | --- | --- |
| CSP | 9.9 | 1.2 | 2.9 | **<0.0001** | **0.02** |
| AMA1-3D7 | 90.3 | 25 | 26 | **<0.0001** | 0.81 |
| EBA-140 | 9.4 | 2.8 | 5.9 | **<0.0001** | **0.003** |
| EBA-175 | 26 | 2.2 | 5.1 | **<0.0001** | 0.12 |
| EBA-181 | 11.4 | 1.4 | 3.9 | **<0.0001** | **0.008** |
| MSP1 | 45.8 | 8.9 | 35.3 | **<0.0001** | **<0.0001** |
| MSP3 | 14.2 | 1.5 | 4.5 | **<0.0001** | **0.005** |
| MSP7 | 11 | 1.8 | 3.9 | **<0.0001** | 0.11 |
| MSP9 | 6.8 | 3.5 | 6.3 | **<0.0001** | **<0.0001** |
| MSP DBL1 | 40.8 | 7.9 | 12.8 | **<0.0001** | **0.02** |
| MSP DBL2 | 76.5 | 11.1 | 23.4 | **<0.0001** | **0.01** |
| Rh5 | 4.5 | 1.3 | 3.2 | **<0.0001** | **0.002** |
